# ASSESSING ADEQUACY OF HEALTH FACILITIES FOR OLDER ADULTS IN INDIAN CITIES

**DOI:** 10.1093/geroni/igad104.3048

**Published:** 2023-12-21

**Authors:** Sruthi Anilkumar Hemalatha, Nawaj Sarif, Papai Barman

**Affiliations:** International Institute for Population Sciences, Mumbai, Maharashtra, India; International Institute for Population Sciences, Mumbai, Maharashtra, India; International Institute for Population Sciences, Mumbai, Maharashtra, India

## Abstract

1The healthcare system in India is struggling to meet the needs of the current population, and the rising aging population is likely to exacerbate this problem. Therefore, it is essential to plan and prepare for the future. Thus, using a geospatial approach, this study used data from the Census of India to investigate the distribution of older populations’ access to improved health facilities across various cities in India. The study found wide variation in the proportion of the aging population across India, with cities in the northern, southern, and eastern parts of India having a higher percentage of older people. The study compared the distribution of older adult populations and health infrastructures, such as hospitals and medical staff, and found considerable regional variations in their availability. Smaller cities tend to have poorer health facilities than larger ones, despite having similar proportions of older populations. The study found that good health facilities were concentrated in specific pockets, particularly in metropolitan cities, while smaller cities lacked them. This infrastructure gap poses a significant challenge in achieving healthy aging in India. Population aging is a significant demographic trend that requires attention from policymakers, researchers, and healthcare professionals. This study highlights the need for improved health facilities in Indian cities, particularly smaller cities, to ensure the well-being and a better quality of life of older adults. By prioritizing the needs of the aging population, we can create a more equitable and sustainable healthcare system that meets the needs of all citizens.

